# Alternative Splicing and Hypoxia Puzzle in Alzheimer’s and Parkinson’s Diseases

**DOI:** 10.3390/genes12081272

**Published:** 2021-08-20

**Authors:** Eglė Jakubauskienė, Arvydas Kanopka

**Affiliations:** Institute of Biotechnology, Life Sciences Center, Vilnius University, Sauletekio al. 7, LT-10257 Vilnius, Lithuania; egle.jakubauskiene@bti.vu.lt

**Keywords:** neurodegenerative diseases, Alzheimer’s disease, Parkinson’s disease, alternative pre-mRNA splicing, splicing factors, hypoxia

## Abstract

Alternative pre-mRNA splicing plays a very important role in expanding protein diversity as it generates numerous transcripts from a single protein-coding gene. Therefore, alterations lead this process to neurological human disorders, including Alzheimer’s and Parkinson’s diseases. Moreover, accumulating evidence indicates that the splicing machinery highly contributes to the cells’ ability to adapt to different altered cellular microenvironments, such as hypoxia. Hypoxia is known to have an effect on the expression of proteins involved in a multiple of biological processes, such as erythropoiesis, angiogenesis, and neurogenesis, and is one of the important risk factors in neuropathogenesis. In this review, we discuss the current knowledge of alternatively spliced genes, which, as it is reported, are associated with Alzheimer’s and Parkinson’s diseases. Additionally, we highlight the possible influence of cellular hypoxic microenvironment for the formation of mRNA isoforms contributing to the development of these neurodegenerative diseases.

## 1. Introduction

Alternative splicing of precursor messenger RNA (pre-mRNA) is one of the most important co-transcriptional and post-transcriptional regulatory mechanisms responsible for the regulation of gene expression and generation of proteomic and functional diversity [1,2,3]. Alternative splicing enables the formation of multiple mRNA variants from a single gene; these mRNA encoded proteins may have distinct structures, functions, stability, or cellular localization [4,5]. The abundance of alternative splicing events reaches >95% in human genes [6], and the main site of alternative splicing events is the brain. Changes in alternative splicing pattern can detrimentally effect the tightly regulated system of the gene expression program and can lead to a large number of human diseases with a predilection for neurological disorders [7].

Neurodegenerative diseases become increasingly prevalent with the aging of the general population. Unfortunately, aging represents the primary risk factor for the development of most neurodegenerative diseases [8]. Several other factors, including neurotoxic effects, genetic predisposition, or traumatic brain injury, also contribute to the progression of neuropathology [9,10]. The widespread neurodegenerative diseases are Alzheimer’s disease, Parkinson’s disease, Pick’s disease, Huntington’s disease, frontotemporal dementia, amyotrophic lateral sclerosis, spinocerebellar ataxia, and motor neuron diseases. Neurodegeneration results in progressive degeneration and/or death of nerve cells that cause progressive loss of memory and various cognitive functions, including lowered performance in attention, language, and movement [5,11].

Alzheimer’s disease (AD) is characterized by an aggregation of β-amyloid (Aβ) plaques and neurofibrillary tangles, which present in patients with progressive cognitive impairment and memory deficits [7]. Alzheimer’s disease is the most common form of dementia that accounts for between 60 and 80% of cases globally [12]. It is considered that the pathogenesis of AD is related by a combination of genetic and nongenetic factors. Genetically, this disease can be familial or sporadic and early or late onset [13]. Most patients (~95%) appear to be sporadic and usually with late onset age (older than 65 years). Nongenetic factors, such as brain trauma, type 2 diabetes, environmental factors, and others, also contribute to progression of sporadic AD [13]. Despite tremendous progress in the knowledge of the molecular pathogenesis of AD, the complete understanding of the reasons for development of AD remains elusive [7].

Parkinson’s disease (PD), the second most common neurodegenerative disease, is associated with selective degeneration of nigrostriatal dopaminergic neurons, although other neurotransmitter systems (i.e., glutamatergic, cholinergic, tryptaminergic, noradrenergic, adrenergic, serotoninergic, and peptidergic) also appear to be affected [14]. It is stated that ~50% of the nigral neurons must degenerate to produce a symptom complex consisting of tremor, rigidity, postural instability, and bradykinesia. In addition to genetic risk factors, environmental toxins (drugs, pesticides) have also been linked to an increased development of PD. Most patients are thought to result from a combination of variable environmental factors on an individual’s composite genetic susceptibility [15,16].

It is reported that the aberrations in the control of gene expression may contribute to the initiation and progression of neurodegeneration (Twine et al., 2011). Current research suggests that pre-mRNA splicing is heavily altered in Alzheimer’s and Parkinson’s diseases [17,18].

## 2. Alternative Pre-mRNA Splicing

**Figure 1:**
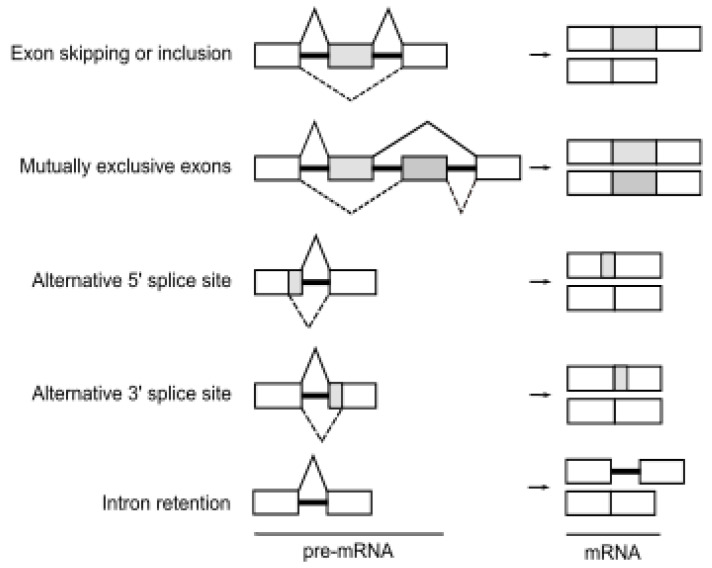
The events of alternative splicing. Various combinations of exons can be spliced to form multiple mRNA and protein products from a single gene. Boxes represent exon sequences, black lines represent intronic sequences. White boxes indicate constitutively spliced exons, gray boxes indicate alternatively spliced regions.

Alternative splicing is an essential step of gene expression that increases transcriptomic and proteomic diversity in eukaryotic cells [19]. In humans, more than 95% of pre-mRNAs are spliced in a developmental, tissue-specific, or signal transduction-dependent manner [3]. In alternative splicing, certain exons are either included or skipped, resulting in various forms of mature mRNA. Several different types of alternative splicing, such as exon skipping or inclusion, alternative 5′ splice sites, alternative 3′ splice sites, mutually exclusive exons, and intron retention are described (Figure 1). Splicing is catalyzed by the spliceosome, a dynamic complex of five small nuclear ribonucleoproteins (U snRNPs) and by more than a few hundred regulatory proteins [20].

**Figure 2:**
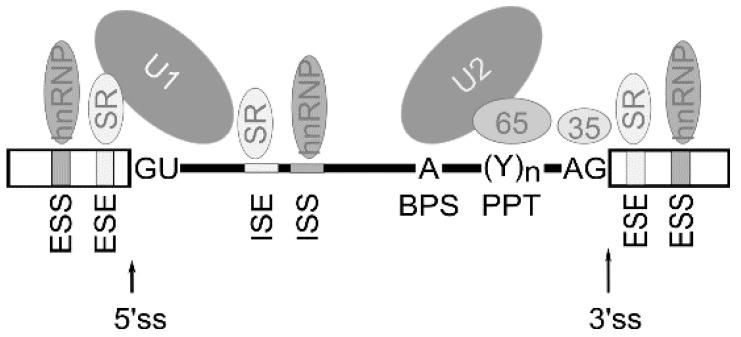
Schematic representation of cis-acting elements and trans-acting factors that regulate splicing. Generally, exonic/intronic splicing enhancers (ESE/ISE) are bound by SR proteins that enhance the splicing, whereas exonic/intronic splicing silencers (ESS, ISS) are bound by hnRNPs that can antagonize the positive effect of SR proteins and inhibit splicing from nearby splice sites. The box indicates exon sequence and the line indicates intronic sequence. ss—splice site, BPS—branch point adenosine, PPT—polypyrimidine tract.

Alternative splicing is known to be regulated by a complex process in which cis-acting elements and trans-acting splicing factors are involved [3]. Cis-acting elements include exonic/intronic splicing enhancers (ESE/ISE) and silencers (ESS, ISS) within the pre-mRNA. Trans-acting factors include the well-characterized splicing regulators are serine–arginine-rich (SR) family proteins, the auxiliary factor of U2 small nuclear ribonucleoprotein (U2AF), heterogeneous ribonucleoproteins (hnRNPs), etc. Thus, multiple splicing factors, such as SR proteins and hnRNPs, regulate exon removal or its inclusion into forming mRNA through binding exonic/intronic splicing enhancers or exonic/intronic splicing silencers in the pre-mRNA [21] (Figure 2).

Alternative pre-mRNA splicing is particularly important in the brain. The brain cells have an unusually high fraction of alternatively spliced genes, thus showing the most complex pattern of alternative splicing and producing a higher diversity of protein isoforms compared to other tissues [22,23]. Changes in alternative splicing result in the formation of different mRNA isoforms in cells and lead to altered gene expression profiles linked to various disease states. Mutations in cis-acting elements or trans-acting factors, alterations in expression, or modification of the level of splicing factors induce changes in splicing pattern.

## 3. Alternative Splicing of Genes Associated with Neurologic Conditions

Changes in alternative splicing of pre-mRNA is increasingly recognized as a driving force in many neurodegenerative diseases. The transcriptome analysis of healthy people and patients allowed the identification of specific splicing changes of AD and PD diseases [24,25], but the role of these changes for diseases development are not well understood. Here, a few examples of alternative splicing genes associated with neurologic disorders are outlined.

**Figure 3:**
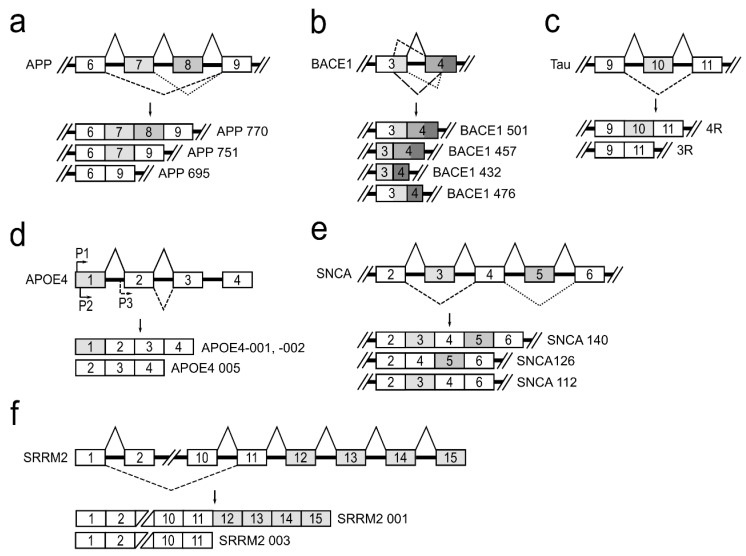
A schematic depiction of alternatively spliced genes contributing to neurodegeneration. (**a**) Alternative APP pre-mRNA splicing forms three major isoforms, APP770, APP751, and APP695, in neurons. (**b**) Alternative BACE1 pre-mRNA splicing and four RNA transcripts: BACE1 501, BACE1 457, BACE1 432, and BACE1 476. (**c**) Alternative splicing of tau exon 10 leads to formation of tau isoforms with three (3R) or four (4R) microtubule-binding repeats. (**d**) Alternative promoter usage for the APOE4 gene generates APOE4-001, -002, and -005 isoforms. (**e**) Three splice variants, SNCA 140, SNCA 126, and SNCA 112, of alternative SNCA pre-mRNA splicing. (**f**) Alternative SRRM2 pre-mRNA splicing produces the longer SRRM2 001 and the shorter SRRM 003 transcripts.

APP is a type I transmembrane glycoprotein, which is ubiquitously expressed, but is the most abundant in the brain [26]. The *APP* gene located on chromosome 21 in humans consists of 18 exons. APP pre-mRNA via an alternative splicing mechanism is joined to produce 11 different mRNA isoforms. In neurons, there are three major isoforms that are important to the development of neurodegenerative diseases: APP770—produced by joining all 18 exons; APP751—isoform lacking exon 8, which codes OX-2 antigen domain; and APP695—isoform lacking exons 7 and 8 that code Kunitz-type protease inhibitor and OX-2 antigen protein domains [27] (Figure 3a). It has been reported that, in the brains of patients with Alzheimer’s disease, APP770 and APP751 mRNA and protein levels are increased and APP695 is reduced [28,29]. Alterations in APP pre-mRNA splicing are associated with increased neurotoxic amyloid-β (Aβ) formation and its accumulation directly promotes AD progression [5]. It is reported that treatment of neuronal cells with estradiol leads to an increased expression of APP695, SC35, and hnRNPA1 and to a reduced level of secreted Aβ. It is suggested that alternative splicing of the *APP* gene is regulated by hnRNPs—a family of splicing factors [30]. Several studies have also demonstrated that alternatively spliced *APP* gene transcripts, including exons 7 and 8, are variably expressed in different regions of the AD brain [31].

Β-site amyloid precursor protein cleaving enzyme 1 (BACE1) is a transmembrane aspartic protease responsible for cleavage of the APP to generate β-amyloid peptide (Aβ) [32]. The *BACE1* gene, located on chromosome 11, is composed of 10 exons and its pre-mRNA undergoes alternative splicing. The use of normal splice sites results in the formation of the full-length active protein BACE1 501. The use of an alternative 5′ splice site within exon 3 and/or an alternative 3′ splice site within exon 4 generates three alternatively spliced transcripts: BACE1 476, BACE1 457, and BACE1 432 (Figure 3b). It is reported that proteins translated from these alternatively spliced transcripts dramatically reduce β-secretase’s activity compared to the full-length BACE1 501 isoform [33]. This fact shows the importance of alternative BACE1 splicing in effecting of Aβ formation and leading to prevention of AD development. Although it is evident that changes in BACE1 splicing can influence Aβ production in cells [34], the protein factors responsible for the regulation of BACE1 alternative splicing, causing the lowering of β-secretase activity, are unknown [35,36,37].

The human tau protein is encoded by the *MAPT* gene located on chromosome 17. *MAPT* gene contains 16 exons. Alternative splicing of exons 2, 3 and 10 in tau pre-mRNA produces six different possible tau mRNAs isoforms that are expressed in the adult human brain [38]. Tau protein binds to microtubules through a microtubule-binding domain comprising four microtubule-binding repeats. Exon 10 encodes the second microtubule-binding repeat motif of tau and consequently, alternative splicing of exon 10 leads to formation of tau isoforms with three (3R) or four (4R) microtubule-binding repeats (Figure 3c). The tau 4R isoform has a higher affinity for microtubule assembly than tau 3R [39]. It is shown that approximately equal levels of tau 4R and 3R isoforms are expressed in adult human brain under normal physiological conditions, and this balance is crucial for the maintenance of neuronal function. However, dysregulation of tau exon 10 splicing leads to disruption of the balance between tau 4R and 3R, and it is sufficient to cause neurodegeneration [40,41,42,43]. It is reported that a family of SR proteins are involved in the regulation of alternative tau exon 10 splicing. Each SR protein influences tau alternative splicing differentially: SRSF1, SRSF2, SRSR6, and SRSF9 promote exon 10 inclusion, while SRSF3, SRSF4, SRSF7, and SRSF11 inhibit its inclusion [44].

Changes in apolipoprotein E gene (*APOE*) also influence the progression of neuropathology [45,46]. APOE protein plays a central role in lipid metabolism in the central nervous system [47]. The *APOE* gene is polymorphic in human populations, with three common alleles (E2, E3, and E4). APOE4 allele is a major AD risk factor, which increases β-secretase (BACE1) processing of APP protein to produce Aβ peptides [48]. APOE4 gene, residing on chromosome 19, is known to undergo alternative splicing and alternative transcriptional promoter usage, generating five isoforms. Three different transcriptional isoforms, APOE4-001, -002, and -005, are expressed in the temporal lobe (Figure 3d). APOE4-001 and -002 contain all four exons, whereas an alternative promoter upstream of exon 2 generates the APOE4 mRNA isoform lacking exon 1. Analysis of the temporal lobe of AD brains revealed that the relative abundance of APOE4-001 is significantly reduced. The expression of dominant APOE4-002 isoform is slightly lowered and isoform APOE4-005 expression is significantly increased [45]. Nevertheless, a full understanding of how the various APOE mRNA isoforms impact brain tissue and which splicing factors are involved in this gene splicing regulation currently remains unknown [17,49].

The accumulation of misfolded presynaptic protein α-synuclein (SNCA) is the main neuropathological hallmark of Parkinson’s disease. The *SNCA* gene maps to chromosome 4 and contains six exons. Three different mRNA isoforms of α-synuclein (α-synuclein 112, α-126, and α-140) are produced by alternative SNCA pre-mRNA splicing (Figure 3e). The α-synuclein 140 isoform includes the entire transcript of the *SNCA* gene. In PD patients, α-synuclein 126 had markedly lower expression, suggesting that it is an aggregation-preventing isoform. However, α-synuclein 112 seems to enhance aggregation under pathological conditions. In the normal brain, equilibrium is maintained among all three transcripts. Therefore, it has been hypothesized that a shift in these ratios may be involved in PD pathogenesis [31,50]. The regulators of α-synuclein pre-mRNA splicing are currently not established.

The serine/arginine repetitive matrix 2 (*SRRM2*) plays an important role in pre-mRNA splicing as a spliceosome component [51]. The *SRRM2* gene generates two main alternative splicing transcripts different at their 3′ end (Figure 3f). The full-length isoform SRRM2 001 contains 15 exons, while the shorter isoform SRRM2 003 lacks exons 12–15. These two mRNA isoforms, as reported, are differentially expressed in PD brain regions. In the substantia nigra of PD, the longer SRRM2 001 mRNA was downregulated, whereas the shorter transcript was upregulated [18,19]. There are no scientific data on mechanisms involved in this gene splicing regulation.

It is also known that mutations in splicing important cis-acting elements in AD associated genes, namely presenilin 1 (PSEN1) [52], presenilin 2 (PSEN2) [53], progranulin (*GRN*) [54], peptidylprolyl cis/trans-isomerase NIMA-interacting 1 (*PIN1*) [55], and PD related genes, such as MAO-B [16] and PARK2 [18], result in the formation of mRNA isoforms, from which translated proteins influence neurological diseases’ development [18].

The presented examples produce convincing evidence that disrupting the relative abundance of alternatively spliced RNA isoforms can lead to the development of the disease [56]. It should also be taken into account that neurodegenerative diseases are linked to an altered cellular microenvironment—hypoxia, which is often named the master regulator of alternative splicing [57].

## 4. Hypoxia, Splicing, and Neurodegeneration

Hypoxia is defined as the state in which oxygen supply is insufficient to the cells and tissues of the body [58]. The cell, in response to hypoxia, induces transcription of a network of target genes. This process is mediated by hypoxia-inducible transcription factors (HIFs). HIFs are heterodimeric transcription factors of the bHLH family composed of α and β subunits. Until now, three α subunits (HIF-1α, HIF-2α, HIF-3α), which function as oxygen sensors, are known. HIF-1β, also called aryl hydrocarbon receptor nuclear translocator protein (ARNT), is constitutively expressed. During normoxia, HIF-1α protein is expressed, but rapidly degraded by the ubiquitin–proteosome pathway. Under hypoxic conditions, HIF-1α protein becomes stabilized and accumulates in the cytosol. Then, HIF-α is translocated into the nucleus where it binds to HIF-β subunit. The HIF heterodimer complex leads to transactivation of HIF target genes involved in response to pathological conditions [59]. It is established that more than 150 genes, affecting angiogenesis, glucose metabolism, apoptosis, and invasion/metastasis are activated by HIF-1 [60]. From a medical point of view, hypoxia is an important pathophysiologic component of many cardiovascular and pulmonary disorders and cancer [61].

A striking change has been observed in alternative splicing pattern of genes and alterations in splicing factor expression under pathologic conditions. It is reported that in human liver cell line hypoxic conditions influence more than 3000 alternative splicing events in more than 2000 genes [60,62,63,64].

Hypoxia-driven alternative splicing changes are mostly studied in cancer, but the brain, as with any other tissue, also requires an uninterrupted supply of oxygen, which is necessary for maintenance of cellular homeostasis and energy metabolism. Consequently, it is not surprising that the deprivation of oxygen triggers a disruption of cellular homeostasis in the brain and causes neuronal cell damage. Hypoxic conditions in cells are known to have a significant effect on the expression of proteins involved in an extensive range of biological processes, such as energy metabolism, erythropoiesis, angiogenesis, neurogenesis to mitochondrial trafficking, and autophagy [65].

It has been shown that the development of neurodegenerative diseases is associated with hypoxic microenvironment in the cell. Respiratory problems are a common feature of these diseases [13,66,67,68,69]. Evidence indicates that hypoxia induces the progression of AD through Aβ aggregation, tau hyperphosphorylation, blood–brain barrier dysfunction, and impaired calcium homeostasis [65,70,71]. It demonstrated that the significant involvement of hypoxia in the processes of neuron degeneration, where hypoxia upregulates β-site APP cleaving enzyme BACE1 gene transcription leading to increased BACE1 secretase activity and production of neurotoxic amyloid β peptide [66,72].

Currently, not much is known about hypoxia-induced alternative splicing impact on AD and/or PD diseases development. In a recent study, it was observed that cellular hypoxic microenvironment promotes exon 10 inclusion into forming tau mRNA, i.e., 4R tau mRNA isoform, which promotes an increase in protein aggregation and formation [73]. It is reported that the dysregulation of tau 3R/4R mRNA isoform balance in the brain cells is sufficient to cause neurodegeneration and dementia [41]; this finding highlights the importance of hypoxia on the development of these diseases.

As splicing factors are the central modulator of alternative splicing, consequently, the alterations in the amount or activity of many splicing factors play a role in synthesis of aberrant splice variants. For example, altered splicing factor SRSF1 cellular expression level changes the formation of tau 3R/4R mRNA splice variants in hypoxic cells [73]. It is known that splicing factors, such as SR proteins, are phosphorylated by kinases that significantly influence their activity [74,75]. It was shown that expressions of protein kinases, such as SRPK1, SRPK2, and CLK1-4, are enhanced in hypoxic including brain cells [73,76,77]; thus, it is not surprising that the tau protein phosphorylation level is increased in hypoxic cells.

A recent report revealed that splicing factor U2AF, due to an increase in phosphorylation level of both subunits, which influence the factor’s interaction with RNA, plays an important role in hypoxia-dependent splicing regulation of Fas pre-mRNA [78]. As neurodegenerative diseases, such as AD and PD, are associated with hypoxic conditions, it is still an open question whether splicing factor U2AF is involved in splicing regulation of neurodegeneration related genes under hypoxic conditions in brain cells.

## 5. Conclusions

The described findings complement the picture of hypoxia influence on the development of neurodegenerative disorders. It is clear that the cellular hypoxic microenvironment via changes in pre-mRNA splicing promotes a generation of different mRNA isoforms and products synthesized from these mRNAs, which are needed for cell adaptation to changed surrounding conditions. Additionally, it is possible that there are more genes associated with neurodegenerative diseases for which splicing depends on cellular hypoxic conditions. Future studies are needed to identify more changes in mRNAs’ isoform expression pattern in brain cells caused by hypoxic conditions and what the impact of these changes is for the development of neurodegenerative diseases.

## Data Availability

Not applicable.
